# Supplementary material for: Diversity of Endophytic Microbes in Taxus yunnanensis and Their Potential for Plant Growth Promotion and Taxane Accumulation
Source: Microorganisms. 2023 Jun 23;11(7):1645. doi: 10.3390/microorganisms11071645 (PMC10383522; doi:10.3390/microorganisms11071645)
Supplement: Supplementary file 1 [file microorganisms-11-01645-s001.zip › microorganisms-2425293-supplementary.pdf]

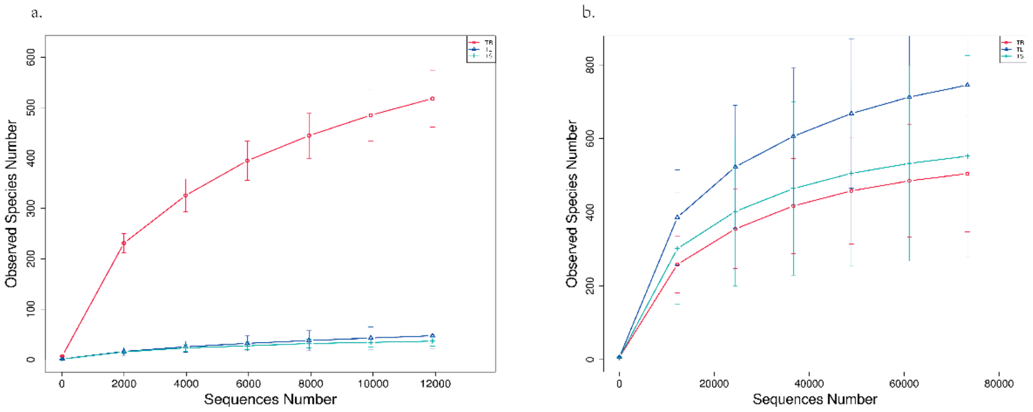

Supplementary Figure S1. Sequencing sample dilution curve. The dilution curve is to randomly select a certain amount of sequencing data from the sample, count the number of species they represent (the number of OTUs), and construct the curve with the amount of sequencing data extracted and the corresponding number of species. (a) Sample dilution curve of endophytic bacteria. (b) Sample dilution curve of endophytic fungi.

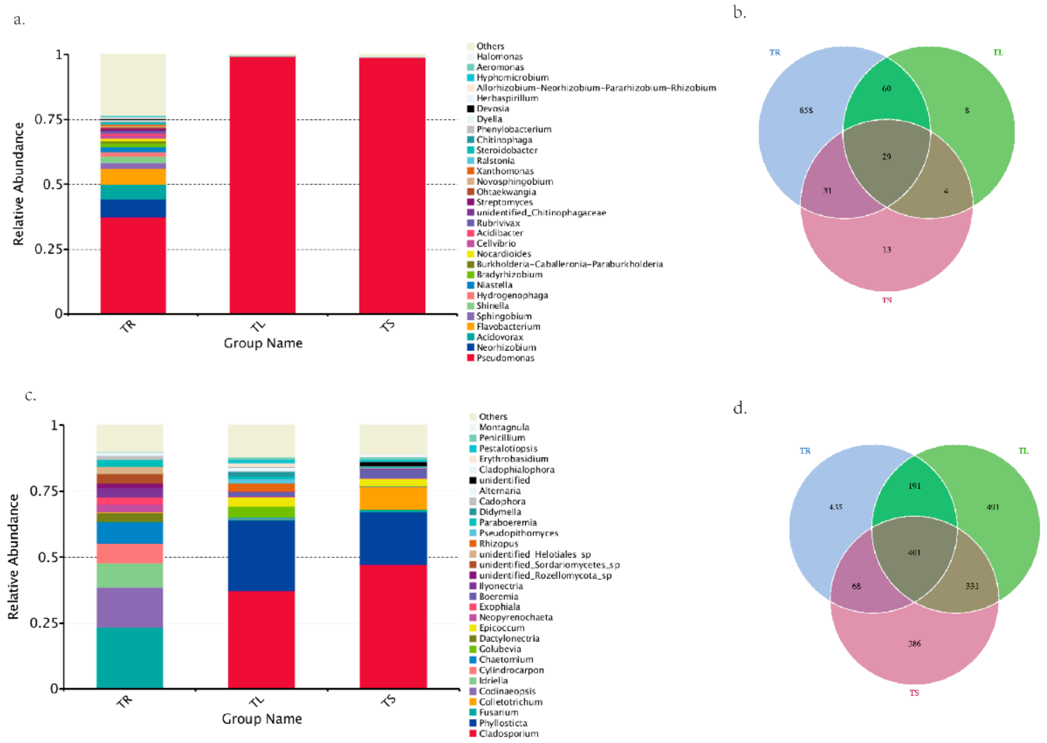

Supplementary Figure S2. The relative abundance histogram of species at the genus level and the Venn Graph based on the number of OTUs in each tissue. (a) Species relative abundance histogram at genus level of endophytic bacteria. (c) Species relative abundance histogram at genus level of endophytic fungi. The abscissa is the sample name; the ordinate represents the relative abundance; others represents the sum of the relative abundance of all the gates other than these 10 gates in the figure. (b) The Venn Graph of endophytic bacteria based on OTU in each tissue. (d) The Venn Graph of endophytic bacteria based on OTU in each tissue. Each circle in the graph represents a set of

samples. The number of circles and overlapping parts of circles represents the number of OTUs shared between the sample groups, and the number of numbers without overlapping parts represents the number of unique OTUs in the sample group.

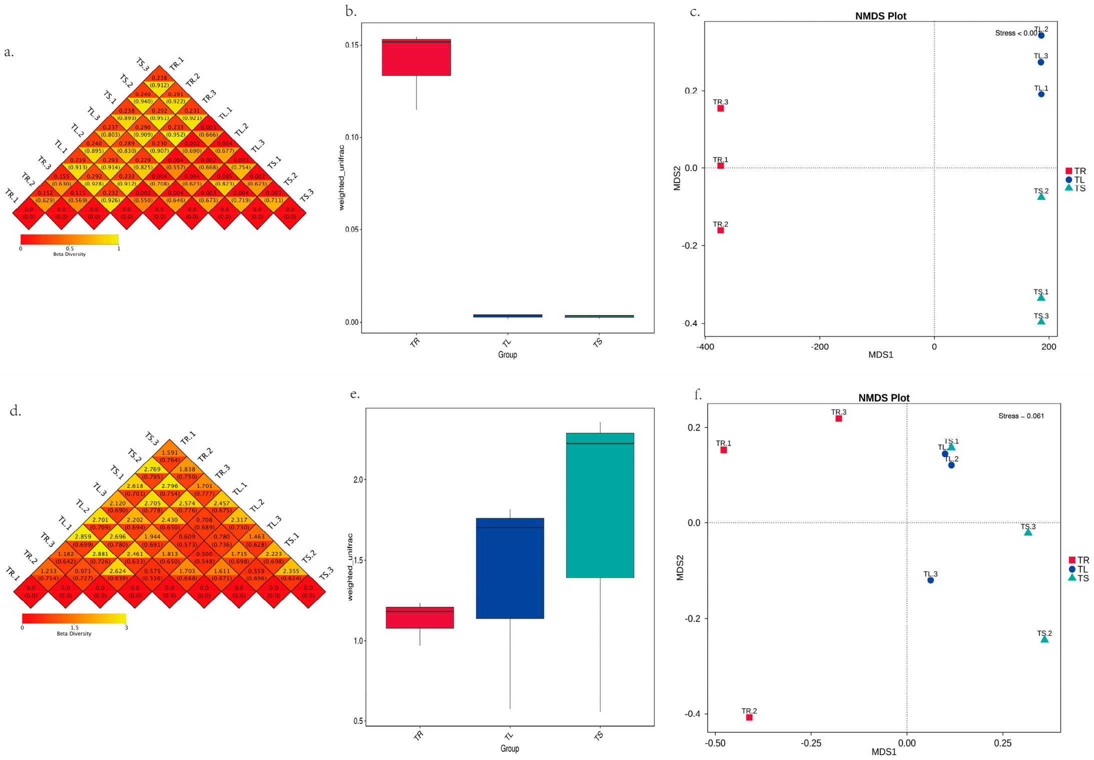

Supplementary Figure S3. Beta Diversity analysis of endophytic bacteria in roots, stems and leaves of *Taxus yunnanensis*. (a) and (d) Beta diversity index heat map. (a) endophytic bacteria. (b) endophytic fungi. The number in the square of the plot is the dissimilarity coefficient between the two samples. The smaller the dissimilarity coefficient, the smaller the difference in species diversity. In the same square, the upper and lower values represent Weighted Unifrac and Unweighted Unifrac distance respectively. (b) and (e) Beta diversity index difference analysis box plot between groups. (b) endophytic bacteria. (e) endophytic fungi. (c) and (f) NMDS (Non-Metric Multi-Dimensional Scaling) analysis of endophytic bacteria in different parts. (c) endophytic bacteria. (f) endophytic fungi. TR, root. TL, leaf. TS, stem.

Table S1. Data preprocessing and quality control statistics.

Sample Name	Raw PE	Raw Tags	Clean Tags	Effective Tags	Base(nt)	AvgLen(nt)	Q20	Q30	GC%	Effective%
TL.1	91,359	90,029	89,174	86,838	19,309,270	222	98.8	96.67	48.62	95.05
TL.2	78,487	77,837	77,178	74,762	16,764,859	224	99.11	97.45	49.47	95.25
TL.3	83,013	82,849	81,715	80,065	22,002,596	275	98.71	95.98	53.62	96.45
TR.1	93,544	90,807	90,251	77,855	16,854,208	216	99.22	97.82	46.55	83.23
TR.2	87,087	85,678	85,261	84,251	18,320,414	217	99.3	97.93	50.95	96.74
TR.3	87,183	86,228	85,498	83,536	18,696,597	224	99.17	97.58	49.93	95.82
TS.1	83,356	81,313	80,783	77,406	17,096,842	221	99.17	97.66	49.76	92.86
TS.2	91,554	90,113	89,570	88,537	19,741,883	223	99.18	97.62	51.02	96.7
TS.3	95,482	94,562	93,779	91,722	21,640,752	236	98.78	96.94	57.1	96.06

Note:Raw PE represents the original PE reads. Raw Tags are spliced Tags sequences. Clean Tags are sequences filtered by Raw Tags with low quality and short length. Effective Tags is a Tags sequence that is used for subsequent analysis after filtering chimeras. Base is the final base number of Effective Data. AvgLen is the average length of Effective Tags. Q20 and Q30 are the percentages of bases with base mass values greater than 20 (sequencing error rate less than 1%) and 30 (sequencing error rate less than 0.1%) in Effective Tags. GC (%) represents the content of GC bases in Effective Tags. Effective (%) represents the percentage of the number of Effective Tags to the number of Raw PEs. TL, leaf samples, TR, root samples, TS, stem samples.
